# Identifying and understanding factors that affect the translation of therapies from the laboratory to patients: a study protocol

**DOI:** 10.12688/f1000research.23663.2

**Published:** 2020-10-16

**Authors:** Manoj M. Lalu, Joshua Montroy, C. Glenn Begley, Tania Bubela, Victoria Hunniford, David Ripsman, Neil Wesch, Jonathan Kimmelman, Malcolm Macleod, David Moher, Alvin Tieu, Lindsey Sikora, Dean A. Fergusson

**Affiliations:** 1 Clinical Epidemiology Program, Ottawa General Hospital Research Institute, Ottawa, Ontario, Canada; 2 Department of Anesthesiology and Pain Medicine, Ottawa Hospital, Ottawa, Ontario, Canada; 3 Department of Cellular and Molecular Medicine, University of Ottawa, Ottawa, Ontario, Canada; 4 BioCurate Pty Ltd, Parkville, Victoria, Australia; 5 Faculty of Health Science, Simon Fraser University, Burnaby, British Columbia, Canada; 6 Department of Medicine, University of Ottawa, Ottawa, Ontario, Canada; 7 Biomedical Ethics Unit, McGill University, Montreal, Quebec, Canada; 8 Center for Clinical Brain Sciences, University of Edinburgh, Edinburgh, UK; 9 Health Sciences Library, University of Ottawa, Ottawa, Ontario, Canada

**Keywords:** Translational failures, promising therapies, bench-to-bedside research, systematic review, case-control study

## Abstract

**Background:** The process of translating preclinical findings into a clinical setting takes decades. Previous studies have suggested that only 5-10% of the most promising preclinical studies are successfully translated into viable clinical applications. The underlying determinants of this low success rate (e.g. poor experimental design, suboptimal animal models, poor reporting) have not been examined in an empirical manner. Our study aims to determine the contemporary success rate of preclinical-to-clinical translation, and subsequently determine if an association between preclinical study design and translational success/failure exists.

**Methods:** Established systematic review methodology will be used with regards to the literature search, article screening and study selection process. Preclinical, basic science studies published in high impact basic science journals between 1995 and 2015 will be included. Included studies will focus on publicly available interventions with potential clinical promise. The primary outcome will be successful clinical translation of promising therapies - defined as the conduct of at least one Phase II trial (or greater) with a positive finding. A case-control study will then be performed to evaluate the association between elements of preclinical study design and reporting and the likelihood of successful translation.

**Discussion:** This study will provide a comprehensive analysis of the therapeutic translation from the laboratory bench to the bedside. Importantly, any association between factors of study design and the success of translation will be identified. These findings may inform future research teams attempting preclinical-to-clinical translation. Results will be disseminated to identified knowledge users that fund/support preclinical research.

## Introduction

**Figure 1.**
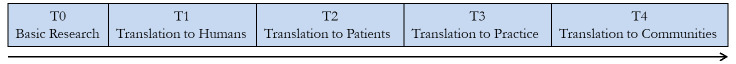
The translational research spectrum.

Advances in discovery research form the backbone for the development of novel therapeutics. Such translation relies on a “process of turning observations in the laboratory, clinic, and community into interventions that improve the health of individuals and the public” ^1^. The process is often visualized as a linear model ( Figure 1). In reality, however, it comprises a spectrum that masks the complex, iterative and interdisciplinary research and development steps that engage multiple stakeholders ^2, 3^. As a result, the successful translation of promising preclinical discovery research into human studies (T1) is rare ^4^, and the resource-intensive efforts to evaluate discoveries in sufficient detail to allow them to be available for patients (T2) takes decades rather than years ^5^.

While discovery research has inherent value, funding agencies around the world are increasingly focused on improving the success of “bench to bedside” translation (T0-T2), thereby enhancing the potential impact of biomedical research budgets. Certainly, there is great potential for improvement; it is estimated that the US alone invests 28 billion dollars per year on research that cannot be reproduced ^6^. Importantly, a clinically translated therapy – one which has obtained regulatory approval – does not mean patients will have access to it. An additional hurdle in the translation into practice (T3) and community (T4) access is whether or not insurance companies and healthcare systems will pay for the therapy. The willingness to pay is largely dependent on a cost-comparative analysis of the potential therapy as well as the needs and resources of the jurisdiction. As this is a critical aspect in the translation spectrum and widespread use of a therapy, it has been thoroughly investigated ^7^.

Investigation of translation in the earlier phases of the translational spectrum – from basic research (T0) to clinical trials in humans (T2) – has uncovered striking deficits when moving from one phase to another ^8^. These inefficiencies ultimately contribute to a 5–10% rate of successful translation of preclinical “bench” research to approved therapies. This means that approximately 90% of promising discoveries may not directly contribute to improved human health ^5, 9, 10^. Moreover, translation failures expose clinical research participants to potential harms of investigational products that fail on safety or efficacy grounds ^11, 12^. The extent of translational failures diverts scarce funding away from developing interventions that are more likely to benefit patients. Given the recent focus on research waste ^13– 16^, it is abundantly clear that bench-to-bedside translation needs solutions to become more efficient.

Three highly cited studies investigating this issue in publicly-available data have been previously published. Contopoulos-Ioannidis *et al*. ^17^ traced the translation of published, highly promising *in vivo* basic science findings into clinical applications from 1979 to 1983 in six basic science journals. They identified 101 promising therapies and in 2003 (i.e. ~20 years since studies published), 27 entered clinical trials and only 5 were clinically licensed (i.e. only 5% translated into an approved therapeutic). Hackam and Redelmeier ^10^ searched for studies published in seven leading journals between 1980 and 2000 that were cited >500 times and investigated highly promising preclinical agents. Their inclusion criteria resulted in 76 studies of interventions, of which 42 (55%) entered clinical trials and only 8 (10%) were approved for clinical use. These authors attempted multivariable analyses to identify predictors of translation; however, the small sample size “limited power to discern individual predictors of translation.” In the most recent investigation of this issue, Morris *et al*. ^18^ (2011) performed a pseudo-systematic search to identify studies that had quantified time lags in the development of health interventions. They found time lags of ~20 years for interventions to translate, though this depended on how each study defined if translation occurred. Importantly, they concluded that the “knowledge of time lags is of limited use to those responsible for R&D…who face difficulties in knowing what they should or can do to reduce time lags.” In other words, identifying factors associated with successful translation of preclinical research into clinical trials may be a more practical metric than time lags for decision makers.

In considering this previous research, it is still currently unknown if/how rates of translation from preclinical research to the therapy’s evaluation in clinical trials are associated with study rigor (i.e. internal validity, risk of bias), and external validity (i.e. reproducibility over a range of conditions), among other factors affecting the quality of the preclinical evidence such as construct validity (i.e. the experimental model appropriately represents the clinical condition and setting). Therefore, the aim of this project is to use established knowledge synthesis methods to identify the prevalence of translation from preclinical research (bench) to the first clinical evaluations of efficacy (bedside), map contemporary trends in translation, and identify key modifiable factors associated with successful and unsuccessful bench-to-bedside translation.

## Objectives

### Objective #1

We will perform a cross-sectional study to estimate the rate of publicly available bench-to-bedside translation. Specifically, we will identify published preclinical ^1^ research of promising therapies and track how far along the translational spectrum they have progressed.

Regulatory approval of the therapy will be considered successful translation. If approval has not yet been obtained, we will consider a therapy that translated from the preclinical phase (T0) to a Phase II clinical trial (T2) or further, a successful bench-to-bedside translation.

### Objective #2

We will next perform a case-control study to identify factors associated with therapies that have been successfully translated from the preclinical study in which it was identified, to a positive clinical trial evaluating treatment efficacy. Specifically, we will consider a therapy to be ‘successfully’ translated if it 1) has obtained regulatory approval, or 2) has a significant, positive finding in a clinical trial evaluating efficacy (presumably a Phase II trial) at the latest stage trial the therapy has advanced to (i.e. the therapy will not be considered successful if it demonstrated efficacy in an earlier trial and subsequently showed null or negative findings in a later efficacy trial).

## Methods

### Protocol

Any deviation(s) from this protocol will be documented in the manuscript that reports the results of this study, upon its completion

### Objective #1

The first objective of this study is to estimate the rate of translation from preclinical research in animal models to success in phase II clinical studies of therapeutic efficacy. This will be achieved through a cross-sectional study of preclinical investigations. In order to identify and select relevant studies for inclusion, we will employ methods typically used in systematic reviews ^19^. The objective focuses on two questions. *What is the current rate of preclinical to clinical translation of promising basic science findings? What are the basic characteristics and design features of the promising therapies identified?*

***Eligibility criteria.*** We will identify preclinical studies published in *Science, Nature,* and *Nature Medicine*. These journals were selected because they are considered leaders across all domains of preclinical research and are anticipated to publish work that may impact human health. Our selection also reflects the philosophy adopted by the largest previous evaluation of bench-to-bedside research ^5^.

Eligible articles will be those published between 1995 and 2015. This timeframe allows a significant time-lag (although not necessarily the full ~20-year time lag described previously). This timeframe will also allow for an evaluation of (in an exploratory manner) whether bench-to-bedside translation has improved in terms of both time-lags and the proportion of therapies advancing to later phases of clinical trials or gaining regulatory approval. Importantly, we anticipate that the journal selection and year range will also provide an adequate sample size to perform the case-control study outlined in Objective #2.

*Inclusion criteria*

i. *Population:*

Articles that describe a preclinical, interventional study: any article that includes *in vivo* non-human animal experiments that has not been tested for the same purpose in humans prior to the study.

ii. *Intervention and outcome:*

A ‘promising therapy’, defined by the following:

a. Any therapy introduced to the animal model (i.e. pharmacologic and non-pharmacologic therapies, vaccines, antibodies, blood products, implants/devices, etc.), which was still at the developmental stage and did not have a prior application in humans for the specific indication.
b. Novel uses of existing therapies (e.g. treatment of a different disease) will also be included.
c. The intervention must induce an outcome that positively benefits an indicator of health of the animal (e.g. an immunotherapy that shrinks solid tumor size in the animal model) and/or investigators state that the therapy should be translated clinically based on their findings.

iii. *Comparison:*

Any comparison to the intervention will be acceptable

*Exclusion criteria*

i. Editorials, commentaries, reviews, news articles, articles that focus on a mechanism of action, pathophysiology, or diagnosis, and articles on agricultural or veterinary applications that would not be feasibly applied to humans.
ii. Investigators explicitly state that translation should not be attempted based on the study.
iii. *Ex vivo, in vitro*, and human clinical trials.

***Information sources and search strategy.*** All included journals are indexed in MEDLINE, therefore this database will be searched from January 1 ^st^, 1995, to December 31 ^st^, 2015. A validated animal filter will limit results to animal studies ^20^. The search strategy will be developed and finalized with the help of an information specialist who has expertise in the design of systematic searches (L. Sikora). The search strategy can be found in *Extended data* ^21^.

***Study selection process.*** The literature search results will be uploaded to Distiller Systematic Review Software (DistillerSR ^®^, Evidence Partners, Ottawa, Canada). DistillerSR is an audit-ready, cloud-based software program that allows for transparent and reproducible work required for an accurate review. Two reviewers will independently screen the titles and abstracts from the search results using the predefined eligibility criteria. A calibration exercise will be performed on the first 50 studies to refine the screening question prior to formally commencing the screening process. Two review authors will assess the eligibility of the full-text articles. Discrepancies between the reviewers will be resolved by discussion or with a third-party member if consensus cannot be established. Reasons for excluding studies will be recorded.

***Data collection process and data items.*** Standardized forms designed in DistillerSR will be used to extract all study characteristics (see list below). Following a pilot to refine the forms and a calibration exercise to ensure high inter-rater agreement (i.e. above 80%), data will be extracted independently by two reviewers. Disagreements between reviewers will be resolved by discussion or with a third-party member if a consensus cannot be reached. The following data will be collected from eligible preclinical articles, and many of these elements will be incorporated for analyses listed in Objective #2. Data items i–v will be collected from all eligible preclinical articles. Data items vi–x will only be collected from articles selected as either cases or controls in Objective #2:

i. *Funding:* academic/governmental/charitable vs. biotechnology and/or pharmaceutical companies (defined as reported industry affiliation by an author, financial support, or provision of the therapy being studied)
ii. *Study characteristics:* study title, first and corresponding authors’ name, publication year, journal of publication, country of corresponding author, and the number of authors and affiliated institutions
iii. *Study population:* animal species, sex, age, presence of comorbid illnesses
iv. *Type of model:* disease being studied, name of animal model(s) used, number of different models used
v. *Interventions (promising therapy):* name of intervention, dose-response tested, anticipated application (e.g. preventative, therapeutic, or both)
vi. *Preclinical outcomes:* primary outcome (or, if none declared, main *in vivo* outcome highlighted by authors), death, adverse events
vii. *Risk of bias (internal validity):* the Cochrane Risk of Bias tool to evaluate preclinical *in vivo* studies ^19^
viii. *External validity* (range of experimental conditions tested) *:* more than one animal model used (species or disease model/co-morbid model), inclusion of male and female animals, and multiple treatment doses
ix. *Completeness of reporting:* an operationalized version of the National Institutes of Health Preclinical Reporting guidelines will be used ^22^.
x. *Article metrics:* The H-index of the corresponding author(s) and the number of times the paper has been cited

***Identifying clinical translation of promising bench findings.*** After identifying the set of promising potential therapies, the stage of clinical translation that each therapy has achieved will be identified. In collaboration with an information specialist (L. Sikora), a piloted algorithmic search strategy that identifies the current state of clinical use will be used. Backward citation analysis (identifying and examining the studies cited in an article) and forward citation analysis (identifying studies that cite an original article or work after it had been published) will be used. The search strategy consists of searching known databases for citation analysis (i.e. Scopus, ISI Web of Science, Google Scholar). Other resources will also be searched including grey literature sources, clinical trial registries, clinical practice guidelines, and conference proceedings. The full draft algorithm is listed in *Extended data*. This approach will be used to determine the furthest stage of clinical testing and whether the agent has received regulatory approval.

***Outcomes.*** The primary outcome of interest will be the successful clinical translation of the identified promising therapies defined as 1) having obtained regulatory approval for the therapy, or 2) the conduct of at least one Phase II trial (or greater) for efficacy, with a statistically significant result favoring the treatment (and no later trials with negative or null results). As per the Federal Drug Agency (FDA) in the United States, the purpose of Phase II trials is “efficacy as well as side effects.” ^23^ A positive finding will be defined as a statistically significant result demonstrating therapeutic superiority compared to placebo/no treatment, or established interventions; or stated non-inferiority compared to currently established interventions in an appropriate designed and conducted clinical study. This primary outcome has been selected as it indicates replication of the findings from the animal models of the preclinical study in the human subjects of the clinical trial.

The secondary outcome is the furthest clinical advances of all promising therapies from Phase I through to regulatory approval. For this outcome we will consider *any* clinical trial that has been performed and/or published. Whether favorable results were obtained for each identified trial (which we will call a “positive” trial) will also be reported. As above, a positive result will be defined by a statistically significant result demonstrating superiority compared to placebo/no treatment, or established interventions; or stated non-inferiority compared to currently established interventions. In determining regulatory approval status, while the dominant regulatory agencies are often regarded as the FDA (US), European Medical Agency (Europe), and Pharmaceuticals and Medical Devices Agency (Japan), approval by any regulatory agency will be accepted. This outcome will be presented as a proportion of studies that advanced to the clinical trial stage. Results will also be stratified by year of publication: 24–20 years ago (to provide a comparison to the landmark article by Contopoulos-Ioannidis *et al*. ^5^), 20–10 years ago, and <10 years ago.

***Data synthesis and analysis plan.*** The total number of studies screened, assessed for eligibility, and included in the review (with reasons for exclusion) will be reported according to the PRISMA guidelines for reporting systematic reviews ^24^. For each included study, the characteristics outlined above will be described, with frequencies and proportions reported. Additionally, we will calculate the proportion of successful Phase II trials that received regulatory approval. For the primary outcome, the time from the preclinical paper publication date, to first positive Phase II trial will be calculated and presented. Likewise, for the secondary outcomes, the time to furthest advance (Phase I, II, III, IV, or approval), as well time to first positive trial will be calculated using medians and interquartile ranges. Kaplan-Meier curves will also be constructed for all time to event analyses. All analyses will be performed using SAS (Version 9.4).

For all promising therapies, a descriptive analysis of all study characteristics and its research trajectory (i.e. furthest level of human experimentation) will be presented. This will present the cohort of successful and unsuccessful promising therapies published in highly influential journals, as well as the ability to evaluate secular trends in translation.

***Risk of bias assessment.*** Risk of bias will be assessed in duplicate by two independent reviewers as high, low or unclear for six domains of bias identified by the Cochrane Risk of Bias tool ^19^, with the addition of two domains suggested to influence the risk of bias in preclinical studies ^25^. Disagreements will be resolved first by discussion and subsequently by consulting a third-party member, if needed. Graphic representations of risk of bias within and across studies will be conducted using RevMan 5.3 (Cochrane Collaboration, Oxford, United Kingdom).

### Objective #2

A case-control study will be performed to evaluate the association between preclinical study characteristics and the incidence of translation. Translated therapies gaining regulatory approval or into a Phase II clinical trial (or later) will be defined as cases; unsuccessfully translated therapies will serve as controls. This study will help identify factors of internal and/or external validity, which are associated with “failed” *versus* “successful” translation. A case-control design was chosen as the most efficient methodological design due to i.) the rarity of preclinical to clinical translation and ii.) the ability to study multiple factors that may contribute to translational success.

***Data source, identification and matching of cases and controls.*** Upon completion of the cross-sectional study outlined in Objective #1, a comprehensive roster of promising basic science findings published in top-tier journals between 1995 and 2015 will be generated. From this dataset, cases and matched controls will be selected:

*Cases:* Promising discoveries that became successfully translated will be used as cases in our study. ‘Successfully translated’ will be defined in two ways as mentioned above. In keeping with the primary outcome in Objective #1, successful translation will be defined as having 1) obtained approval from a regulatory agency and/or 2) demonstrated positive findings in a Phase II clinical trial of efficacy - indicating replication of the findings from the preclinical study. As in Objective #1, a positive result will be defined by a statistically significant result demonstrating superiority compared to placebo/no treatment, or established interventions; or stated non-inferiority compared to currently established interventions.

*Controls:* Highly promising basic science findings which were not successfully translated will serve as controls. Unsuccessful translation will be defined as either a highly promising basic science finding without 1) having obtained regulatory approval, AND 2) any clinical trial (i.e. no trial identified by literature search and/or identified in clinical trial registries such as clinicaltrials.gov) or going to trial and failing for any reason (e.g. failing in safety in Phase I; or efficacy in Phase II). If possible, up to 4 matched controls per case will be selected. This 4:1 ratio represents the most statistically efficient ratio while minimizing bias introduced to the study ^26, 27^. Controls will be frequency matched based on year of publication (+/- 5 years for each case, disease of interest, and general area of biomedical research (i.e. cardiology, cancer, immunotherapy, etc.)). These stratifying factors are not predictor variables but have been chosen to ensure the comparability of cases and controls in the sample. Following matching, controls will be randomly selected using a computer-generated algorithm for those cases with >4 matched controls.

***Variables.*** The primary analysis will investigate the association between a set of variables thought to influence translation, and successful translation. It is hypothesized that the following four sets of variables are of high importance:

i. *Internal validity:* Similarly to clinical studies, preclinical studies that lack methodological rigor usually demonstrate the largest measures of efficacy ^28– 30^. However, this lack of methodological rigor may reduce the likelihood that these studies can be translated. In our study, risk of bias will be assessed by applying the Cochrane Risk of Bias tool ^19^ – modified for suitability to preclinical studies. We will assess six domains identified by this tool (sequence generation, allocation concealment, blinding of personnel, blinding of outcome assessors, incomplete outcome data, and selective outcome reporting), as well as two additional risk of bias domains (conflict of interest and sample size calculation) for a total of eight risk of bias domains. Again, this checklist will be operationalized into a binary outcome; if the majority (i.e. >4) of items on the checklist are met the study will be considered to have high internal validity.
ii. *External validity:* This will be assessed by the experiment(s) within the preclinical study being performed across a range of conditions: the presence of dose response relationship, more than one animal model (species or method of disease induction) used, male and female animals (or the appropriate sex for sex specific diseases, e.g. prostate or ovarian cancer), and co-morbid animals ^31^. This outcome will be dichotomized, (i.e. if two or more of these four criteria are met, the study will be considered to have high external validity).
iii. *Funding source:* Evidence suggests that industry involvement may lead to faster translation ^5^. This relationship will be examined by determining if the preclinical study involving the promising therapy was industry, government, or philanthropy funded (with attention also given to whether industry funding was from pharmaceutical or biotechnology companies).
iv. *Completeness of reporting:* The National Institutes for Health preclinical reporting guidelines will be used to assess completeness of reporting. We have previously operationalized this list into 21 yes or no questions. This outcome will be dichotomized as high or low completeness of reporting when the majority of items (≥11) are reported upon or not. As a pre-planned sensitivity analysis, we will also evaluate the preclinical studies as high completeness of reporting when they report on over two thirds of the checklist items (≥14).

For the exposure items of validity (internal and external) and reporting the individual items in each checklist will not be weighted.

*Exploratory analysis:*

i. Individual components of internal validity: We will assess two selected risk of bias domains (sequence generation and blinding of outcome assessor) individually.

ii. Individual components of external validity: We will assess all components of external validity individually, rather than as an aggregate outcome.

iii. *Number of authors and institutions:* We will use the number of co-authors and the number of affiliated institutions on the preclinical paper as an approximation for the degree of collaboration within the preclinical study. With this, we plan to evaluate how the degree of collaboration within a preclinical study affects translation.

iv. *H-index:* We will use the corresponding author’s H-index as an approximation for investigator seniority. With this, we plan to evaluate how the corresponding author’s seniority affects preclinical to clinical translation.

v. *Number of citations:* We will use the number of citations associated with the preclinical paper as an approximation for the degree of dissemination. With this we plan to evaluate how the degree of the preclinical study’s dissemination affects translation.

***Data analysis and sample size***

**Sample size.** In a preliminary screen of *Science* and *Nature*, 482 promising therapies (184 Science; 298 Nature) were identified. Application of a conservative estimate suggests that the addition of the third journal ( *Nature Medicine*) will provide an additional 150 promising therapies, for a total of 632. In the previous report citing a 5% translation rate ^17^, 19 of 101 (19%) promising therapies reached the clinical trial stage and demonstrated favorable results. Methodological differences between this study and the previous report may lead to a decrease in the percentage of promising therapies demonstrating favorable results at phase II. A conservative estimate of half the rate from the previous report (9.5%) suggests that roughly 60 of the promising therapies from our sample would demonstrate promising results at phase II or later. Generally, it is recommended that a regression model contain 10 events per variable ^32, 33^. Considering the four variables listed above, we will require 40 cases in order to be adequately powered to perform our proposed analysis.

If an adequate sample size of 40 cases is not reached, we will continue searching for promising preclinical therapies in order to identify additional phase II or later translated therapies. The CAMARADES group has used a machine learning algorithm ^34^ to identify a cohort of over 100,000 reports of *in vivo* research available on PubMed Central. From this we will randomly select reports of *in vivo* preclinical research and assess these for clinical translation. The number of studies randomly selected will depend on the number of additional case required. We will sample iteratively until our predetermined minimal set of 40 cases is obtained.

**Analytic plan.** A comparison of baseline variables (aside from matching factors) in the cases and controls will be assessed using frequency distributions and univariate descriptive statistics including measures of central tendency and dispersion. Multivariable conditional logistic regression will be performed to determine the adjusted association of our factors to the outcome variable. Odds ratios with accompanying 95% confidence intervals will be calculated. A sensitivity analysis will be performed excluding cases that had a positive clinical trial but had not received regulatory approval. All analyses will be performed using SAS (version 9.4). Data will be presented as odds-ratios and 95% confidence intervals in forest plots.

### Limitations

Our focus on leading journals ( *Science*, *Nature* and *Nature Medicine*) may be perceived as a limitation. Given the usually positive findings and higher visibility of studies published therein, this may lead to an over-estimate in the bench-to-bedside translation. Although this selection of journals may represent an over-estimate of translation, we also believe it represents the ‘best-in-class’ of preclinical research. Alternatively, the reliance upon publicly available data may result in an under-estimate of the bench-to-bedside transition as it is possible that preclinical work from within industry that does not proceed to a clinical trial may not be published. Without examining this omitted data, we may not be presenting a fully accurate assessment of the state of preclinical to clinical trial translation. Furthermore, our sample is limited to three journals, which may reduce the scope of our work. As automation of meta-research projects gains traction (and tools are validated) broader searches will certainly be possible in the future. However, given that this process currently relies on personnel we are limited to the current strategy. Another limitation is that components of internal validity have historically been poorly reported in bench research ^35^, which may not be an accurate reflection of what investigators actually did. A third limitation is that the dichotomization of variables in the case-control could be regarded as an oversimplification of complex concepts. However, our exploratory analyses will allow us to investigate a larger number of factors in more granular detail. Furthermore, our assessment on the external validity and how this may affect translation rests on the assumption that the rationale for initiating a clinical trial is based solely on one preclinical study, rather than several studies from different labs under a range of conditions. In reality, external validity would be best established through multiple preclinical experiments rather than one with a high level of external validity. 

Another important aspect affecting the quality of preclinical evidence is construct validity. Studies have demonstrated that poor construct validity affects reproducibility and downstream clinical translation ^36, 37^. Though a checklist to assess construct validity has been developed ^31^, after pilot testing this tool we found that it was infeasible for our study, as it required content expertise for every promising therapeutic to be used appropriately. Thus, another limitation of this study is that we are unable to evaluate the construct validity of the preclinical studies and assess how this aspect affects the translation into clinical trials.

Lastly, it should be acknowledged that the translational spectrum involves many complex steps and various phases. Though ultimate clinical translation is the approval and adoption of a therapy, we will focus on a specific stage in the translational spectrum ^1^. We have chosen to measure the translation of the *in vivo* animal experiment stage within T0 research; to the first evaluation of the therapy’s efficacy in clinical trials, within T2 research. When we deem a successful translation from preclinical to clinical research, we do not evaluate all stages of preclinical nor clinical research. Furthermore, successful translation to a Phase II clinical trial does not imply that the therapy has been successfully translated to practice or regulatory approval; and regulatory approval may not guarantee widespread adoption. With this limitation in mind, we will focus on preclinical issues that may affect early translation, rather than potential issues with the clinical trials that subsequently evaluated the therapies in human subjects or barriers that affect both regulatory approval and adoption into practice We anticipate a future study that will critically analyze the translational clinical trials identified in Objective #1 to address issues associated with those trials.

### Dissemination

We will have a one-day, in-person meeting between the investigators and identified knowledge users (Stem Cell Network and BioCanRx, two Government of Canada funded Networks of Centres of Excellence focused on the development of novel therapeutics). Over the one-day meeting we will examine data and results generated by the project. In addition, each knowledge user will speak about their organization’s experiences and perspectives on bench-to-bedside translation. The meeting will end with a discussion to identify next steps to be taken by stakeholders to improve translation.

We anticipate the publication of two key papers submitted to appropriate peer-reviews journals. The first will describe key findings of the systematic review and present the results of our case-control study. The second will be a policy piece to describe how our findings may affect stakeholders across the spectrum of bench-to-bedside research. The results of this study will be presented at relevant national and international scientific meetings to promote knowledge transfer.

### Amendments

If amendments are required for this protocol, the date of each amendment will be provided with a description for rationale for the change in this section.

## Study status

With our systematic search strategy, we have searched the journals *Science*, *Nature*, and *Nature Medicine* and are currently screening articles to identify promising therapies as per Objective #1 of this study.

## Discussion

This study is an innovative project that will use knowledge synthesis methods to identify the incidence of successful translation, map contemporary trends in translation, and identify key modifiable factors associated with successful and unsuccessful bench-to-bedside translation. Additionally, the proposed study will generate the largest dataset to date of promising preclinical therapies and then apply a case-control design to investigate the association of validities with successful or failed translation. This evidence is required to implement a “knowledge-to-action” ^38– 40^ cycle to improve bench-to-bedside translational research.

Current lags in translation are unknown. Some of our own efforts (and others) at translation suggest that these lags between preclinical findings and first-in-human trials may be shortening ^25, 41– 44^. This reduction may reflect increased funding towards translational activities, improved research rigor, as well as regulatory changes that have accelerated translation efforts (e.g. FDA’s four unique approaches of Fast Track, Breakthrough Therapy, Accelerated Approval, and Priority Review). If this hypothesis is supported by the data, all stakeholders need information about the factors that support improvements in development timelines.

The effect of evolving preclinical reporting guidelines on translation is unknown. In the last decade, funders, journals, and other stakeholders have endorsed reporting guidelines ^35, 45^ that improve transparency and potentially improve the ability to forecast which interventions may translate successfully. These guidelines have been developed in response to multiple publications, which demonstrated that the majority of basic science findings are irreproducible ^46– 48^. Previous studies of bench-to-bedside translation were conducted prior to these guidelines being widely endorsed; thus, a current study is needed to determine if increased awareness and emphasis regarding preclinical reporting and design has improved translation.

Taken together, there is a strong need and justification for the proposed study. This study will provide a contemporary understanding of bench-to-bedside translation, which is required to tailor experimental designs to improve translational efficiency, including the education and uptake of available study design and analysis methods.

## Data availability

### Underlying data

No data is associated with this article.

### Extended data

Open Science Framework: Identifying and understanding factors that affect the translation of therapies from the laboratory to patients, https://doi.org/10.17605/OSF.IO/WGZ3T ^21^.

This project contains the following extended data:

- Representative search strategy in PubMed
- Data selection items
- Algorithm to identify clinical translation of promising bench findings

Data are available under the terms of the Creative Commons Zero “No rights reserved” data waiver (CC0 1.0 Public domain dedication).

## Footnotes

^1^ The term ‘preclinical’ has variable definitions, depending on the context. For the purposes of this study, our group has chosen to focus on preclinical interventional *in vivo* animal experiments, relevant to the betterment of human health and/or treatments for human disease.
