# Supplementary material for: Identifying and understanding factors that affect the translation of therapies from the laboratory to patients: a study protocol
Source: F1000Res. 2020 Oct 16;9:485. Originally published 2020 Jun 1. [Version 2] doi: 10.12688/f1000research.23663.2 (PMC7570319; doi:10.12688/f1000research.23663.2)
Supplement: Peer review history

# Peer review: Reviewer response for version 2

**DOI:** 10.5256/f1000research.30157.r73235

**Published:** 2020-10-22

**Authors:** Jonathan A Lal

**Affiliations:** 1 Department of Molecular and Cellular Engineering, Jacob Institute of Biotechnology and Bioengineering, Sam Higginbottom University of Agriculture, Technology and Sciences, Allahabad, Uttar Pradesh, India

I am satisfied with the responses and updated corrections to my comments. It is now approved from my side.

Is the study design appropriate for the research question?

Yes

Is the rationale for, and objectives of, the study clearly described?

Yes

Are sufficient details of the methods provided to allow replication by others?

Yes

Are the datasets clearly presented in a useable and accessible format?

Not applicable

Reviewer Expertise:

NA

I confirm that I have read this submission and believe that I have an appropriate level of expertise to confirm that it is of an acceptable scientific standard.

# Peer review: Reviewer response for version 2

**DOI:** 10.5256/f1000research.30157.r73234

**Published:** 2020-10-16

**Authors:** John Holcomb

**Affiliations:** 1 Division of Acute Care Surgery, Department of Surgery, University of Alabama at Birmingham, Birmingham, AL, USA

No further comments

Is the study design appropriate for the research question?

Partly

Is the rationale for, and objectives of, the study clearly described?

Yes

Are sufficient details of the methods provided to allow replication by others?

Yes

Are the datasets clearly presented in a useable and accessible format?

Yes

Reviewer Expertise:

injury, resuscitation,

I confirm that I have read this submission and believe that I have an appropriate level of expertise to confirm that it is of an acceptable scientific standard.

# Peer review: Reviewer response for version 1

**DOI:** 10.5256/f1000research.26110.r69847

**Published:** 2020-09-23

**Authors:** Jonathan A Lal

**Affiliations:** 1 Department of Molecular and Cellular Engineering, Jacob Institute of Biotechnology and Bioengineering, Sam Higginbottom University of Agriculture, Technology and Sciences, Allahabad, Uttar Pradesh, India

Reference 4 is too old (from 2006) to claim that translation from preclinical to human trials is rare. This should be updated.

 Reference 6 is 5 years old, should work, but if more recent data is available, perhaps replace.

 Reference 7 is too old to be valid in today's economic development, please update.

 For methods it says any changes will be included in the final report. It is to be noted that the peer review is limited to the manuscript itself.

 Eligibility criteria limited to 3 prominent journals limits the scope as JAMA and other such high impact factor (preclinical/clincal) journals are left out. At least 5 journals could have been taken into consideration to justify the sample size.

 What would be interesting to see is the ratio between successful Phase II trails and those successful Phase II trails which actually reach regulatory approvals.

 Furthermore, regulatory approval may not still guarantee widespread adoption. The latter of which could be due to other factors which remain to be explored, including competition. A comment towards this end would be advised.

 In limitations it mentions preclinical to Phase II, and eventually analysing the preclinical problems till Phase II. Phase III and/or Phase IV excluded, regulatory touched upon, it seems this should be made clear at the start of the manuscript, that the focus is till Phase II and not bedside directly.

Is the study design appropriate for the research question?

Yes

Is the rationale for, and objectives of, the study clearly described?

Yes

Are sufficient details of the methods provided to allow replication by others?

Yes

Are the datasets clearly presented in a useable and accessible format?

Not applicable

Reviewer Expertise:

NA

I confirm that I have read this submission and believe that I have an appropriate level of expertise to confirm that it is of an acceptable scientific standard.

# Response

**Published:** 2020-10-08

**Authors:** Manoj Lalu

**Affiliations:** Ottawa Health Research Institute, Canada

**We thank you for your careful assessment of our protocol and their supportive comments. We have addressed your queries below and within the revised manuscript.**

 Reference 4 is too old (from 2006) to claim that translation from preclinical to human trials is rare. This should be updated. 

**Updated citation to: Seyhan, A.A. Lost in translation: the valley of death across preclinical and clinical divide – identification of problems and overcoming obstacles. transl med commun 4, 18 (2019). https://doi.org/10.1186/s41231-019-0050-7**

 Reference 6 is 5 years old, should work, but if more recent data is available, perhaps replace. 

**Updated citation to: Hutchins BI, Davis MT, Meseroll RA, Santangelo GM (2019) Predicting translational progress in biomedical research. PLoS Biol 17(10): e3000416. https://doi.org/10.1371/journal.pbio.3000416**

 Reference 7 is too old to be valid in today's economic development, please update. 

**We agree with the reviewer that this is an old reference.  We believe this may not have been the strongest argument to present in the introduction, thus we have removed the sentences on return on investments.**

 For methods it says any changes will be included in the final report. It is to be noted that the peer review is limited to the manuscript itself. 

**Changed sentence to: *Any deviation from this protocol will be documented in the manuscript that reports the results of this study, upon its completion.***

 Eligibility criteria limited to 3 prominent journals limits the scope as JAMA and other such high impact factor (preclinical/clincal) journals are left out. At least 5 journals could have been taken into consideration to justify the sample size. 

**Our group of investigators considered this very issue at length. Ultimately, there simply is no perfect sampling strategy to address the objectives of the study. We selected the three journals as they are highly influential and considered ‘best-in-class’ by many. The number of articles resulting from the search of the three included journals is 20,341. With our available resources, it has taken close to one year to screen all abstracts and full texts in duplicate. Adding more journals would not be feasible with our finite resources. We added the following to the limitation section of the discussion:**

***Furthermore, our sample is limited to three journals, which may reduce the scope of our work. As automation of meta-research projects gains traction (and tools are validated) broader searches will certainly be possible in the future.  However, given that this process currently relies on personnel we are limited to the current strategy.***

 What would be interesting to see is the ratio between successful Phase II trails and those successful Phase II trails which actually reach regulatory approvals. 

**We certainly agree with this suggestion. This is something we will report on in the manuscript that summarizes the results of this study. We added the following sentence in the analysis plan of Objective 1:**

***Additionally, we will calculate the proportion of successful Phase II trials that received regulatory approval*** *.*

 Furthermore, regulatory approval may not still guarantee widespread adoption. The latter of which could be due to other factors which remain to be explored, including competition. A comment towards this end would be advised. 

**We also agree with this comment and have addressed this limitation in our discussion:**

***Furthermore, successful translation to a Phase II clinical trial does not imply that the therapy has been successfully translated to practice or regulatory approval; and regulatory approval may not guarantee widespread adoption. With this limitation in mind, we will focus on preclinical issues that may affect early translation, rather than potential issues with the clinical trials that subsequently evaluated the therapies in human subjects or barriers that affect both regulatory approval and adoption into practice.***

 In limitations it mentions preclinical to Phase II, and eventually analysing the preclinical problems till Phase II. Phase III and/or Phase IV excluded, regulatory touched upon, it seems this should be made clear at the start of the manuscript, that the focus is till Phase II and not bedside directly. 

**We would argue that Phase II is considered bedside as the evaluation is being completed on patients.  However, we do agree that this is quite different from full clinical adoption and therefore should not be conflated with widespread approval. As such we have modified the following in our manuscript:**

***Lastly, it should be acknowledged that the translational spectrum involves many complex steps and various phases. Though ultimate clinical translation is the approval and adoption of a therapy, we will focus on a specific stage in the translational spectrum*** ***^4^*** ***. We have chosen to measure the translation of the in vivo animal experiment stage within T0 research; to the evaluation of the therapies’ efficacy in clinical trials, within T2 research.***

# Peer review: Reviewer response for version 1

**DOI:** 10.5256/f1000research.26110.r69397

**Published:** 2020-08-17

**Authors:** John Holcomb

**Affiliations:** 1 Division of Acute Care Surgery, Department of Surgery, University of Alabama at Birmingham, Birmingham, AL, USA

The authors have embarked on an important project. They will evaluate the issues related to translation of preclinical data into clinical practice.

 I have several comments.

1. The authors should clearly state the narrow focus of their work, largely in the drug development arena. They seem to exclude other research areas such as devices and blood products. Please inform the reader what focus areas are included and excluded.
2. The authors have included only several journals to review. Given the ability to use electronic databases, I would suggest they broaden their search.
3. Lastly, the definition of clinical translation is not completion of phase 2 studies, but rather regulatory approval and adoption into clinical practice. Defining adoption itself is difficult, and should be discussed.  

Is the study design appropriate for the research question?

Partly

Is the rationale for, and objectives of, the study clearly described?

Yes

Are sufficient details of the methods provided to allow replication by others?

Yes

Are the datasets clearly presented in a useable and accessible format?

Yes

Reviewer Expertise:

injury, resuscitation,

I confirm that I have read this submission and believe that I have an appropriate level of expertise to confirm that it is of an acceptable scientific standard, however I have significant reservations, as outlined above.

# Response

**Published:** 2020-10-08

**Authors:** Manoj Lalu

**Affiliations:** Ottawa Health Research Institute, Canada

**We thank you for your careful assessment of our protocol and their supportive comments. We have addressed your queries below and within the revised manuscript.**

 The authors have embarked on an important project. They will evaluate the issues related to translation of preclinical data into clinical practice. I have several comments.

  

1. The authors should clearly state the narrow focus of their work, largely in the drug development arena. They seem to exclude other research areas such as devices and blood products. Please inform the reader what focus areas are included and excluded.

 

**We apologize for the lack of clarity on our inclusion criteria. Our search and strategy for selecting therapies is designed to consider all interventions that have therapeutic benefit.  We added blood products and devices in the examples of ‘promising therapies’ in the inclusion criteria.**

***A ‘promising therapy’, defined by the following:***

***a. Any therapy introduced to the animal model (e.g. pharmacologic and non-pharmacologic therapies, vaccines, antibodies, blood products, implants/devices, etc.), which was still at the developmental stage and did not have a prior application in humans for the specific indication.***

  

1. The authors have included only several journals to review. Given the ability to use electronic databases, I would suggest they broaden their search.

 

**Our group of investigators considered this very issue at length. Ultimately, there simply is no perfect sampling strategy to address the objectives of the study. We selected the three journals as they are highly influential and considered ‘best-in-class’ by many. The number of articles resulting from the search of the three included journals is 20,341. With our available resources, it has taken close to one year to screen all abstracts and full texts in duplicate. Adding more journals would not be feasible with our finite resources. We added the following to the limitation section of the discussion:**

***Furthermore, our sample is limited to three journals, which may reduce the scope of our work. As automation of meta-research projects gains traction (and tools are validated) broader searches will certainly be possible in the future.  However, given that this process currently relies on personnel we are limited to the current strategy.***

  

1. Lastly, the definition of clinical translation is not completion of phase 2 studies, but rather regulatory approval and adoption into clinical practice. Defining adoption itself is difficult, and should be discussed.  

 

**We agree that full clinical translation is not the completion of a Phase II trial and we also do agree that ultimate clinical translation is the clinical adoption of a therapy. In the current study we are focussed on the translation of preclinical research to first studies demonstrating efficacy in clinical trials. Full clinical translation to approved therapies will also be tracked and we will certainly be reporting on this metric as well.**

**We have added/modified a sentence in the manuscript to further explain our intention:**

***T*** ***herefore, the aim of this project is to use established knowledge synthesis methods to identify the prevalence of translation from preclinical research (bench) to the first clinical evaluations of efficacy (bedside),*** ***map contemporary trends in translation, and identify key modifiable factors associated with successful and unsuccessful bench-to-bedside translation*** **.**

**We added the following sentence in the analysis plan of Objective 1:**

***Additionally, we will calculate the proportion of successful Phase II trials that received regulatory approval*** *.*

**We also added and modified the following in our limitations section:**

***Lastly, it should be acknowledged that the translational spectrum involves many complex steps and various phases. Though ultimate clinical translation is the approval and adoption of a therapy, we will focus on a specific stage in the translational spectrum*** ***^4^*** ***. We have chosen to measure the translation of the in vivo animal experiment stage within T0 research to the first evaluation of the therapy’s efficacy in clinical trials, within T2 research.***
